# Specific Amino Acids Affect Cardiovascular Diseases and Atherogenesis via Protection against Macrophage Foam Cell Formation: Review Article

**DOI:** 10.5041/RMMJ.10337

**Published:** 2018-07-30

**Authors:** Claudia Grajeda-Iglesias, Michael Aviram

**Affiliations:** The Lipid Research Laboratory, Rappaport Faculty of Medicine, Technion–Israel, Institute of Technology, Haifa, Israel

**Keywords:** Amino acids, atherogenesis, BCAA, CVD, lipid metabolism, macrophages

## Abstract

The strong relationship between cardiovascular diseases (CVD), atherosclerosis, and endogenous or exogenous lipids has been recognized for decades, underestimating the contribution of other dietary components, such as amino acids, to the initiation of the underlying inflammatory disease. Recently, specific amino acids have been associated with incident cardiovascular disorders, suggesting their significant role in the pathogenesis of CVD. Special attention has been paid to the group of branched-chain amino acids (BCAA), leucine, isoleucine, and valine, since their plasma values are frequently found in high concentrations in individuals with CVD risk. Nevertheless, dietary BCAA, leucine in particular, have been associated with improved indicators of atherosclerosis. Therefore, their potential role in the process of atherogenesis and concomitant CVD development remains unclear. Macrophages play pivotal roles in the development of atherosclerosis. They can accumulate high amounts of circulating lipids, through a process known as macrophage foam cell formation, and initiate the atherogenesis process. We have recently screened for anti- or pro-atherogenic amino acids in the macrophage model system. Our study showed that glycine, cysteine, alanine, leucine, glutamate, and glutamine significantly affected macrophage atherogenicity mainly through modulation of the cellular triglyceride metabolism. The anti-atherogenic properties of glycine and leucine, and the pro-atherogenic effects of glutamine, were also confirmed *in vivo*. Further investigation is warranted to define the role of these amino acids in atherosclerosis and CVD, which may serve as a basis for the development of anti-atherogenic nutritional and therapeutic approaches.

## INTRODUCTION

Cardiovascular diseases (CVD) currently account for nearly half of the non-communicable diseases and remain the leading cause of death worldwide, with 80% of the deaths occurring in low- and middle-income countries, and coronary artery diseases (CAD) and stroke being the most frequent direct causes of death.^1^,^2^ The underlying cause of CVD is atherosclerosis development, a chronic inflammatory disease that arises from an imbalance in lipid metabolism and a maladaptive immune response.^3^ The strong relationship between CVD, atherosclerosis, and lipid metabolism has been recognized for decades, and, although cholesterol deposits within the artery have been thought to initiate atherosclerosis development, cholesterol is not the only circulating lipid that causes the disease. Triglycerides and their major component, fatty acids, are central molecules in lipoprotein metabolism and a major cause of heart dysfunction. Deposition of triglyceride-rich lipoproteins or their remnants within the artery, or vascular cytotoxicity from those lipolysis products, is the supported hypothesis for the relationship between triglyceride levels and vascular diseases.^4^–^7^ Nevertheless, the focus on the lipid–atherosclerosis relationship has overlooked the potential contribution of other key dietary components, such as amino acids, to the process of atherogenesis, and the concomitant development of CVD.

## ROLE OF AMINO ACIDS IN CARDIOVASCULAR DISEASES AND IN ATHEROSCLEROSIS DEVELOPMENT

During the last few years, through the inclusion of comprehensive metabolic profiling, called “metabolomics,” individual or clusters of amino acids have been identified as novel biomarkers and metabolic signatures which are associated with incident cardiovascular disorders, suggesting their significant role in the pathogenesis of CVD.^8^ However, while novel data correlate some specific amino acids with increased CVD risk, these associations do not prove a cause–effect relationship between altered circulating amino acids and cardio-metabolic diseases. In consequence, different and even opposite conclusions are frequently triggered from associative studies based on serum amino acid levels and mechanistic studies that focused on the biological effects of amino acids on the diseases.^9^ For instance, glutamate and glutamine have been associated with atherosclerosis development and CVD risk, as well as related cardio-metabolic disorders. In subjects with cardiac catheterization, levels of glutamate/glutamine were the most significant metabolite discriminator between CAD and non-CAD patients.^10^ High glutamate values were associated with the incidence of coronary heart disease (CHD), independent of traditional risk factors.^11^ Furthermore, glutamine was linked with clinical manifestations of atherosclerosis, since it was found to be associated with increased risk for both plaque development and increased intima–media thickness.^12^ However, the opposite was shown by a recent study in postmenopausal women, which reported glutamine as the only metabolite associated with decreased risk of CHD, while glutamate remained a biomarker after adjustment for traditional CHD risk factors.^13^ Along with these two amino acids, other potential atherogenic amino acids are methionine and its metabolic intermediate, homocysteine, which have been found to promote atherosclerosis development in humans and in animal models. Methionine-induced hyperhomocysteinemia accelerated early plaque development and enhanced plaque fibrosis in susceptible atherosclerotic mice (apolipoprotein E-deficient [apoE^−/−^]), via different mechanisms including impaired anti-oxidant activity, increased lipid peroxidation, and also through enhanced macrophage foam cell formation.^14^–^20^

Regarding amino acid association with decreased CVD risk and anti-atherogenic effects, glycine, the simplest amino acid, has been inversely correlated with the risk of acute myocardial infarction (MI) in patients with suspected angina pectoris.^21^ In addition, arginine, the main precursor for nitric oxide (NO) production in the vascular endothelium, improved endothelial function in CVD or overweight patients.^22^ In line with these associations, orally supplemented glycine or glycine+arginine decreased plasma homocysteine levels, considerably increased total NO concentration, and countered the elevated plasma and hepatic cholesterol-to-phospholipids ratio, in hypercholesterolemic rats. Moreover, glycine was involved in enhancing the availability of NO in the vasculature (by reducing its oxidation in a glutathione-dependent mechanism) and in NO-dependent vasodilatation (by stimulating the *N*-methyl-D-aspartate receptor).^23^,^24^ Additionally, *in vitro* studies reported anti-inflammatory effects of glycine supplementation during endothelial inflammation.^25^ On the other hand, and in contrast to the above anti-atherogenic reports, increased serum levels of arginine were positively associated with the presence of atherosclerotic plaques in a large adult cohort,^26^ emphasizing the existing conflict between correlative and mechanistic studies.^9^ Consistent with this controversy, recent studies have described a strong positive correlation between the levels of plasma branched-chain amino acid (BCAA), including leucine, isoleucine, and valine, and metabolic diseases, and recognized them as biomarkers for CVD risk,^27^ while others claim the potential role of BCAA catabolism in cardiac pathophysiology.^28^,^29^

## BRANCHED-CHAIN AMINO ACID AS BIOMARKERS FOR CVD RISK

Leucine, isoleucine, and valine constitute the group of BCAA, due to shared structural features in their side-chain and a distinct catabolic pathway in the first two steps of their catabolism. Unlike other amino acids, BCAA are primarily catabolized in the extrahepatic tissues, notably the cardiac muscle. The branched-chain aminotransferase converts BCAA into branched-chain α-keto-acids (BCKA), which, eventually, can be oxidized in the liver by BCKA dehydrogenase, the rate-limiting step in the BCAA catabolic pathway, to acetyl-CoA, ketones, and/or intermediates of the tricarboxylic acid cycle.^30^ Branched-chain amino acids are essential for normal growth and function at the cellular and the organ levels. In addition, BCAA, and leucine in particular, can act as signaling molecules through their molecular targets, including mammalian target of rapamycin complex 1 (mTORC1), AMP-activated protein kinase, peroxisome proliferator-activated receptors γ and α (PPARγ and α, respectively), and coactivator-1α (PGC-1α).^31^,^32^ However, an excess amount of free BCAA or their catabolic products can also be cytotoxic.^28^ Elevated concentrations of each or total BCAA were found in individuals with cardiovascular risk factors, such as high fasting blood glucose, dyslipidemia, or increased serum atherosclerosis index (ratio between serum triglycerides and high-density lipoproteins [HDL]), in patients with diagnosed CAD,^8^,^33^,^34^ in men with metabolic syndrome risk,^35^ or in healthy individuals, independently of their BMI.^36^ Moreover, BCAA were shown to be predictors for hypertriglyceridemia in early adulthood.^37^

In two large community-based cohorts, Cheng et al.^38^ observed higher circulating concentrations of BCAA in individuals with metabolic risk factors such as obesity, impaired glucose tolerance, dyslipidemia, or blood pressure. In line with these results, Shah et al.^8^ reported the association of BCAA with mortality, independently of standard predictors, in patients undergoing cardiac catheterization. Ruiz-Canela et al.^27^ recently conducted a case-cohort study including incident CVD cases and demonstrated the significant association of baseline leucine or isoleucine concentrations with higher CVD risk after adjustment for potential confounders, and this correlation was stronger for stroke, in a high cardiovascular risk population. Moreover, their study showed that circulating levels of BCAA may be independent of the amount of BCAA ingested with the diet. Recently published results from a long-term (18.6 years of follow-up) prospective observational cohort of women, free of CVD at baseline, confirmed the positive association of total BCAA with incidence of CVD, which was comparable to the association of low-density lipoprotein (LDL)-cholesterol with CVD.^39^ In spite of these associations, high dietary intake of BCAA, and particularly leucine, the major BCAA with an important cardio-metabolic role, was associated with improved measures of cardio-metabolic biomarkers, including dyslipidemia,^40^ direct measures of arterial stiffness, such as pulse wave velocity and intima–media thickness, or atherosclerosis development,^41^ in healthy women, independently of genetic confounding.

An essential role for BCAA catabolism for normal cardiac physiology and cellular viability has been demonstrated through experimentation in murine heart failure (HF) models, suggesting a defective BCAA catabolism as a metabolic hallmark of failing heart. The BCAA-impaired catabolism resulted in accumulation of BCKA, which directly suppressed respiration and induced superoxide production in isolated mitochondria, thus promoting HF in a mouse model and in human dilated cardiomyopathy heart.^42^ This was associated with induced oxidative stress and metabolic disturbance, and the transcription factor Krüppel-like factor 15 was identified as a key regulator of the BCAA catabolism in the heart.^42^ Accordingly, it was recently found that myocardial BCAA catabolism was significantly impaired in response to permanent MI, leading to an elevation of myocardial BCAA abundance and post-MI cardiac dysfunction.^43^ Importantly, the same study showed that pharmacological enhancement of BCAA catabolism alleviated post-MI cardiac pathologies.^43^ Moreover, Li et al.^44^ found that impaired BCAA catabolism and their subsequent accumulation selectively disrupted mitochondrial pyruvate utilization through inhibition of the pyruvate dehydrogenase complex (PDH) activity, with marked decreases in glucose uptake and oxidation, in glycogen content, and in protein glycosylation, thus rendering the heart vulnerable to ischemia-reperfusion injury. The pyruvate dehydrogenase complex was identified as a key regulatory point through which BCAA modulates cardiac metabolism. In line with these findings, a very recent randomized, controlled trial conducted in in-hospital HF-patients demonstrated that supplementation with oral BCAA significantly improved hypoalbuminemia and the cardiothoracic ratio, which are common features of HF condition.^45^ Moreover, BCAA treatment preserved cardiac function, prolonged survival, and increased gene expression related to mitochondrial biogenesis and function, in skeletal muscles in a HF-rat model.^46^

Therefore, whether BCAA are the cause per se, an epiphenomenon of, or indicators of cardio-metabolic disturbance remains the paramount question.^47^ To investigate this further, interventional studies including supplementation with BCAA have been carried out in cells, in animal models, and in human populations. While BCAA-supplemented blood mononuclear cells showed increased production of reactive oxygen species (ROS) via both NADPH oxidase and the whole mitochondria, stimulating the activation of the redox-sensitive transcription factor NF-κB, which resulted in the release of pro-inflammatory molecules,^48^ BCAA supplementation reduced the expression of interleukin (IL)-6, IL-1β, IL-18, and tumor necrosis factor-α (TNFα) mRNA in the liver, as well as the amount of hepatic triglycerides accumulation, together with inhibition of macrophage infiltration, in the white adipose tissue of obese mice.^49^ Additionally, in mice with choline-deficient high-fat diet-induced non-alcoholic steatohepatitis, BCAA alleviated hepatic lipid accumulation and ameliorated mitochondrial dysfunction in liver, preventing liver injury, through downregulation of hepatic fatty acid synthase (FAS), sterol regulatory element-binding protein-2 (SREBP-2), microsomal triglyceride transfer protein, and an increased citrate synthase activity.^50^ In a similar way, dietary leucine was effective in decreasing hepatic expression of lipogenic proteins (FAS, SREBP-1, LXRa, and acetyl-coenzyme A carboxylase), while decreasing serum levels of pro-inflammatory adipokines (leptin, IL-6, and TNFα), which improved serum and liver lipid profile (total cholesterol and triglycerides) in mice fed a high-fat/high-cholesterol diet.^51^ Accordingly, leucine was found to decrease hepatic triglyceride accumulation in mice with fatty liver.^52^ Furthermore, decreased atherosclerotic lesion area—via improved plasma lipid profile (decreased LDL and very-low-density lipoproteins [VLDL], and increased HDL) and through downregulation of ATP binding cassette transporters G5 and G8, which participate in hepatic cholesterol efflux to the bile—was observed in the atherosclerotic apoE^−/−^ mice upon administration of leucine.^53^ In addition, leucine enhanced the athero-protective effects of nicotinic acid in LDL receptor-deficient (LDLR^−/−^) mice, particularly by reducing aortic infiltration of macrophages.^54^

Nevertheless, although emerging data suggest novel lipid-lowering properties for BCAA, and particularly leucine, their potential role in the early atherosclerosis development remains understudied.^55^ With this in mind, the next step aimed to elucidate the underlying metabolic and molecular mechanisms relating BCAA and leucine in particular to the protection against atherogenesis.

## ROLE OF AMINO ACIDS IN MACROPHAGE FOAM CELL FORMATION, THE KEY FEATURE DURING ATHEROSCLEROSIS DEVELOPMENT

Macrophages are recognized as key pathophysiological agents in wide-spread disease processes associated with chronic inflammation and aging, including atherosclerosis.^56^ A crucial early step in atherosclerosis development is the infiltration of monocytes from the circulation into the arterial wall,^57^ where they differentiate into macrophages and accumulate lipids in a process known as macrophage foam cell formation, the hallmark feature of early atherogenesis.^58^ The accumulation of lipids, notably cholesterol and a substantial amount of triglycerides, in macrophages, their conversion into foam cells, and the initiation and progression of the atherosclerotic lesions are determined mainly by the balance between lipoprotein uptake by macrophages, lipid biosynthesis rate within the macrophages, and the lipid clearance from the macrophage cells, known as cholesterol efflux.^58^–^60^

**Figure 1:**
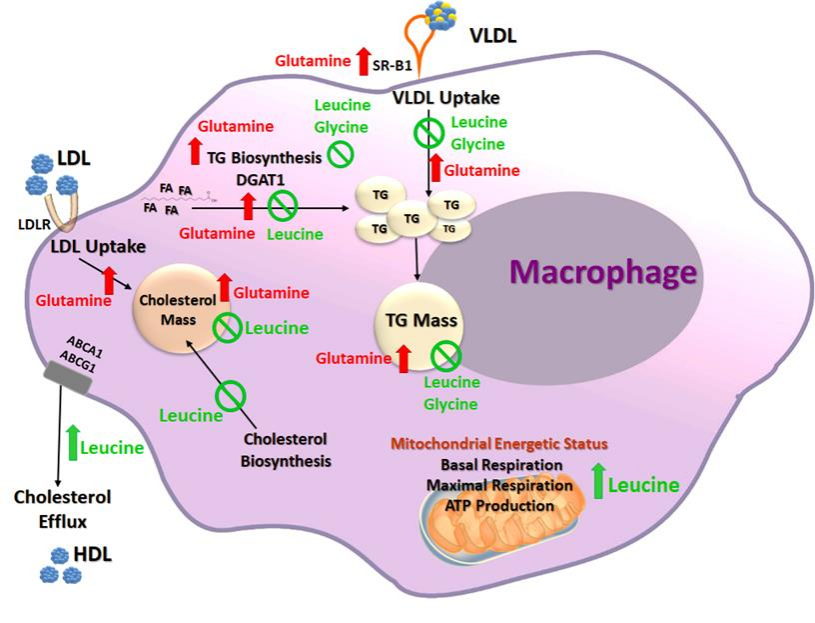
Amino Acids Affect Macrophage Foam Cell Formation through Regulation of Lipid Metabolism Leucine and glycine significantly prevented triglyceride accumulation in macrophages, by inhibiting triglyceride-rich very-low-density lipoprotein (VLDL) uptake and triglyceride biosynthesis rate, while glutamine showed the opposite effects, accompanied by a concurrent upregulation of diacylglycerol acyltransferase-1 (DGAT1). Leucine also decreased macrophage cholesterol content by inhibiting the rate of cholesterol biosynthesis and increasing serum-mediated cholesterol efflux from macrophages, whereas glutamine increased the uptake of cholesterol-rich low-density lipoproteins (LDL), with concomitant accumulation of cholesterol mass. Macrophage mitochondrial respiration and ATP production were improved after leucine supplementation. Red-colored up-arrows (indicating increase or upregulation) and compounds names, designate pro-atherogenic effects; green-colored up-arrows, crossed circles (indicating decrease or inhibition), and compounds names, designate anti-atherogenic effects. ABCA1, ABCG1, ATP-binding cassette subfamily A or G member 1; DGAT1, diacylglycerol acyltransferase-1; FA, fatty acids; HDL, high-density lipoprotein; LDL, low-density lipoprotein; LDLR, LDL receptor; SR-B1, scavenger receptor type B-1; TG, triglycerides; VLDL, very-LDL.

The direct influence of exogenous or endogenous fatty acids on atherosclerosis development has been clearly demonstrated, both scientifically and clinically. In contrast, the contribution of amino acids to the process of macrophage foam cell formation, and the setting of the inflammatory diseases, remains underexplored. Based on the above, we recently evaluated the effects of all 20 amino acids on atherogenesis using murine macrophages. We have analyzed cellular toxicity, generation of ROS, as well as cellular cholesterol or triglyceride content. Through this screening, we were able to identify six specific amino acids, namely glycine, cysteine, alanine, leucine, glutamate, and glutamine, which at non-toxic concentrations significantly affected lipid accumulation in the arterial cells, with a major protective effect on macrophage triglyceride metabolism, given by decreased uptake of the triglyceride-rich VLDL and reduced macrophage triglyceride biosynthesis rate (Figure 1).^55^ We identified glutamate and glutamine as pro-atherogenic compounds, since they stimulated the triglyceride accumulation in macrophages through an enhanced rate of triglyceride biosynthesis, mediated by the induction of key regulators of cellular triglyceride biosynthetic pathways, including SREBP-1, the key regulator of cellular triglyceride biosynthesis,^61^ and diacylglycerol acyltransferase-1 (DGAT1), which catalyzes the final step of this pathway.^62^ Additionally, glutamate and glutamine showed marked stimulatory effects on macrophage oxidative stress and on the overexpression of the scavenger receptor SR-B1, a regulator of macrophage oxidative status and lipid metabolism.^63^,^64^

On the other hand, glycine, cysteine, alanine, and leucine showed clear anti-atherogenic effects, by significantly reducing macrophage triglyceride content, related to decreased VLDL uptake. Glycine was the only amino acid that attenuated both the uptake of the triglyceride-rich VLDL and the triglyceride biosynthesis rate in macrophages, which is in line with the previously reported cardio-protective effect of this amino acid on endothelial cells, thus, strongly suggesting a glycine anti-atherogenic role.^25^,^65^ Further experiments *in vivo* using the atherosclerotic apoE^−/−^ mouse model, supplemented with glycine or glutamine, supported our *in vitro* findings. While glycine supplementation significantly decreased the triglyceride levels in serum and in the macrophages isolated from the mouse peritoneum, glutamine supplementation of apoE^−/−^ mice significantly increased the cellular oxidative stress and the accumulation of cholesterol and triglycerides in macrophages, as observed also in our *in vitro* studies and explained by an increased uptake of LDL and VLDL by macrophages. Furthermore, a trend to decreased aortic content of cholesterol, triglycerides, and lipid peroxides was observed in the glycine-treated mouse group.^55^

We next explored the roles that leucine plays in macrophage lipid metabolism (Figure 1). In addition to decreasing macrophage triglycerides content, leucine also significantly attenuated cholesterol mass in the macrophage model system. In an in-depth investigation supplementing leucine to humans, mice, or cultured macrophages, we clearly demonstrated leucine’s potent *in vitro* and *in vivo* lipid-lowering effects in macrophages.^66^ The above was the first study to report the beneficial effects of leucine on human serum atherogenicity. Murine macrophages were incubated with serum obtained from healthy humans supplemented with leucine, which provoked a significant decrease in macrophage cholesterol mass by inhibiting the cholesterol biosynthesis rate. Furthermore, leucine increased cholesterol efflux from macrophages, which was apparently independent of the anti-atherogenic properties of HDL.^66^ Similarly, cholesterol content in peritoneal macrophages harvested from leucine-supplemented mice was significantly attenuated in relation to reduced cholesterol biosynthesis rate. Additionally, we found a significant decrease in hepatic cholesterol and triglycerides in the leucine-supplemented mice, similar to previously reported data.^50^–^52^ Moreover, our studies in cultured J774A.1 murine macrophages revealed reduced macrophage VLDL uptake and a marked inhibition of triglyceride biosynthesis rate, with concurrent downregulation of DGAT1, after supplementation with leucine at physiological concentrations. Interestingly, we observed similar effects in the macrophages treated with α-ketoisocaproate, the first metabolite derived from leucine catabolism, with a potential cardio-protective role.^67^ Finally, considering previous studies which relate mitochondrial dysfunction to the progression of atherosclerosis *in vivo*,^2^,^68^,^69^ we investigated the effect of leucine supplementation on macrophage mitochondrial energetic status. We observed a significant increase in mitochondrial basal and in maximal respiration, as well as increased mitochondrial ATP production, in both *in vitro* and *in vivo* models, indicating that leucine supplementation indeed improved the mitochondrial energetic status of macrophages, further revealing a new potential athero-protective feature of leucine, although the underlying molecular/biochemical mechanisms still remain unclear.^66^

**Figure 2:**
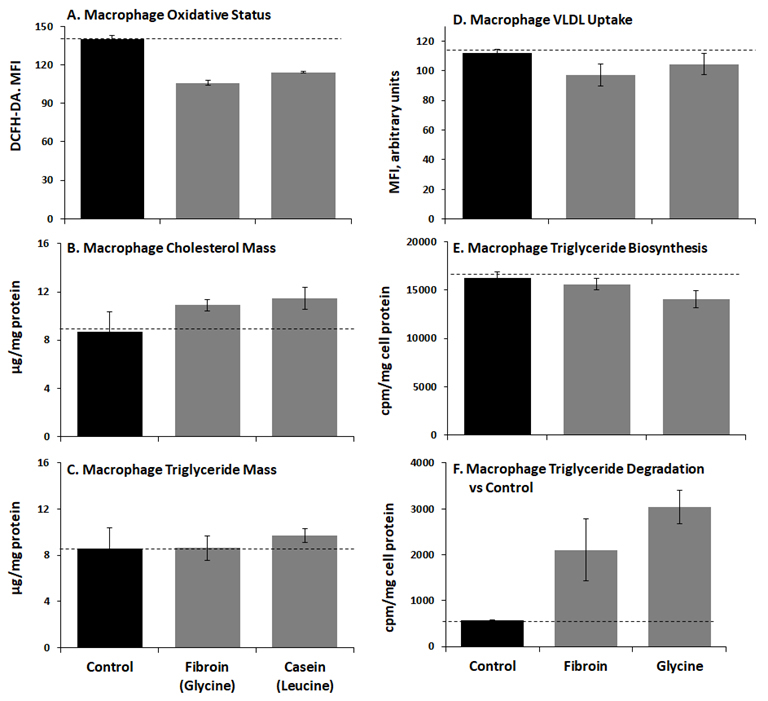
Oxidative Status and Lipid Metabolism upon Addition of Glycine- or Leucine-Rich Proteins (Fibroin or Casein, Respectively) to Cultured J774A.1 Macrophages Quantifications were performed after cell incubation with fibroin (glycine-rich), casein (leucine-rich), or only glycine, followed by: (A) Intracellular ROS generation measured by 2′,7′-dichlorodihydrofluorescein diacetate (DCFH-DA); (B) Cholesterol mass; (C) Triglyceride mass; (D) VLDL uptake, using fluorescein isothiocyanate (FITC)-labeled VLDL; (E) Triglyceride biosynthesis rate after cell incubation with [3H]-oleic acid; (F) Triglyceride degradation rate versus Control.

As leucine and glycine were shown to be the most anti-atherogenic amino acids in our macrophage model system,^55^,^66^ we have recently assessed macrophage supplementation with proteins which are glycine-rich, such as fibroin (from silk worm), or leucine-rich, such as casein,^70^ for their capability to affect macrophage oxidation, cholesterol mass, and triglyceride content (Figure 2). Our preliminary results show that upon addition of the leucine-rich protein (casein) or the glycine-rich protein (fibroin), a decreased oxidative status in J774A.1 cultured macrophages was noted (macrophage oxidation, cholesterol mass, and triglyceride content decreasing by 18%, 24%, and 15%, respectively, Figure 2A). Cellular cholesterol and triglyceride mass were not affected by any of the proteins tested (Figure 2B and C, respectively), whereas macrophage triglycerides metabolism was considerably attenuated, through the observed attenuation in VLDL uptake by macrophages upon using fibroin or glycine (up to 13%, Figure 2D). Furthermore, fibroin and glycine reduced macrophages triglyceride biosynthesis rate (up to 14%) and increased triglycerides degradation rate (by 25% and 36%, Figure 2E and F, respectively). Therefore, this preliminary study using proteins rich in glycine or in leucine supports and emphasizes the significant role of these specific amino acids in the inhibition of macrophage foam cell formation and atherogenesis, as it was previously demonstrated in our *in vivo* studies using individual amino acids (glycine or leucine) supplementation.^55^,^66^

## CONCLUSIONS AND PERSPECTIVES

Atherosclerosis, the major cause of CVD worldwide, is considerably affected by exogenous dietary factors.^71^ While lipids have been designated as the main dietary contributors to the onset of this inflammatory disease, novel data indicated a potential involvement of some specific amino acids in the process of macrophage foam cell formation, the hallmark feature of early atherogenesis. Through a systematic analysis of all 20 amino acids, potential anti- or pro-atherogenic amino acids were identified. For instance, glycine, cysteine, alanine, leucine, glutamate, and glutamine significantly affected macrophage foam cell formation, mainly through modulation of cellular triglyceride metabolism.^55^ Furthermore, an in-depth investigation (Figure 1) conducted in humans, mice, and cultured macrophages revealed that leucine modified macrophage lipid metabolism with a simultaneous enhancement of mitochondrial respiration, suggesting novel metabolic mechanisms by which leucine inhibits macrophage foam cell formation and atherogenesis, strongly supporting previous reports on the beneficial role of leucine on lipid metabolism and its ability to inhibit tissue lipids accumulation, a key feature of both fatty liver diseases and atherosclerosis development.^50^,^51^,^53^–^55^,^66^

Recently, it was suggested that, given their essential role in metabolic homeostasis, the effects of BCAA are largely dependent on the catabolic and anabolic states of the organisms.^72^ A correlation between BCAA and cardio-metabolic disease was found to be age-dependent and was obviously more pronounced in young adults than in the elderly.^73^ It is proposed that under conditions of energy deprivation or homeostasis BCAA may improve glucose uptake/insulin sensitivity. However, under conditions of chronic excess energy, BCAA catabolism is disrupted, causing the frequently observed accumulation of BCAA and their related metabolites, both intracellularly and in the circulation, thus explaining the association between high circulating BCAA values and CVD.^74^ Interestingly, increasing evidence has raised the hypothesis that elevated circulating BCAA may originate from the gut microbiota rather than from dietary sources.^75^ Overall, considering our recent findings, together with the available data so far, it was clearly shown that specific amino acids, mainly BCAA, and especially leucine, play a significant role in atherogenesis protection. Further investigation using tissue-specific knockout mouse models are needed in order to advance our knowledge on amino acid atherogenicity protection by using the above amino acid-rich diet (mostly the BCAA leucine and glycine). Additionally, in the case that gut microbiota particularly contributes to the circulating BCAA concentrations, this might represent a target for the development of cardio-protective and anti-atherogenic therapeutic approaches based on supplementation with specific amino acids, and especially BCAA.

## Abbreviations

ApoE^−/−^: apolipoprotein E-deficient
BCAA: branched-chain amino acid
BCKA: branched-chain α-keto-acids
CAD: coronary artery diseases
CHD: coronary heart disease
CVD: cardiovascular diseases
DCFH-DA: 2′,7′-dichlorodihydrofluorescein diacetate
DGAT1: diacylglycerol acyltransferase-1
FAS: fatty acid synthase
FITC: fluorescein isothiocyanate
HDL: high-density lipoproteins
HF: heart failure
IL: interleukin
LDL: low-density lipoprotein
LDLR^−/−^: LDL receptor-deficient
MI: myocardial infarction
mTORC1: mammalian target of rapamycin complex 1
NO: nitric oxide
PDH: pyruvate dehydrogenase complex
PPAR: peroxisome proliferator-activated receptor
ROS: reactive oxygen species
SREBP: sterol regulatory element-binding protein
TNFα: tumor necrosis factor-α
VLDL: very-low-density lipoproteins.
